# MicroRNAs Associated with Von Hippel–Lindau Pathway in Renal Cell Carcinoma: A Comprehensive Review

**DOI:** 10.3390/ijms18112495

**Published:** 2017-11-22

**Authors:** Lisa-Maria Schanza, Maximilian Seles, Michael Stotz, Johannes Fosselteder, Georg C. Hutterer, Martin Pichler, Verena Stiegelbauer

**Affiliations:** 1 Division of Oncology, Department of Internal Medicine, Medical University of Graz, 8036 Graz, Austria; Lisa-Maria.Schanza@klinikum-graz.at (L.-M.S.); michael.stotz@klinikum-graz.at (M.S.); johannes.fosselteder@edu.uni-graz.at (J.F.); martin.pichler@medunigraz.at (M.P.); 2 Research Unit of Non-Coding RNA and Genome Editing in Cancer, Division of Oncology, Department of Internal Medicine, Medical University of Graz, 8036 Graz, Austria; 3 Department of Urology, Medical University of Graz, 8036 Graz, Austria; maximilian.seles@medunigraz.at (M.S.); georg.hutterer@medunigraz.at (G.C.H.); 4 Department of Experimental Therapeutics, The University of Texas MD Anderson Cancer Center, Houston, TX 77054, USA

**Keywords:** renal cell carcinoma, Von Hippel-Lindau, angiogenesis, microRNAs

## Abstract

Renal cell carcinoma (RCC) are the most common renal neoplasia and can be divided into three main histologic subtypes, among which clear cell RCC is by far the most common form of kidney cancer. Despite substantial advances over the last decade in the understanding of RCC biology, surgical treatments, and targeted and immuno-therapies in the metastatic setting, the prognosis for advanced RCC patients remains poor. One of the major problems with RCC treatment strategies is inherent or acquired resistance towards therapeutic agents over time. The discovery of microRNAs (miRNAs), a class of small, non-coding, single-stranded RNAs that play a crucial role in post-transcriptional regulation, has added new dimensions to the development of novel diagnostic and treatment tools. Because of an association between Von Hippel–Lindau (VHL) genes with chromosomal loss in 3p25-26 and clear cell RCC, miRNAs have attracted considerable scientific interest over the last years. The loss of VHL function leads to constitutional activation of the hypoxia inducible factor (HIF) pathway and to consequent expression of numerous angiogenic and carcinogenic factors. Since miRNAs represent key players of carcinogenesis, tumor cell invasion, angiogenesis, as well as in development of metastases in RCC, they might serve as potential therapeutic targets. Several miRNAs are already known to be dysregulated in RCC and have been linked to biological processes involved in tumor angiogenesis and response to anti-cancer therapies. This review summarizes the role of different miRNAs in RCC angiogenesis and their association with the VHL gene, highlighting their potential role as novel drug targets.

## 1. Introduction

Renal cell carcinoma (RCC) represents the most common renal neoplasia, whereby particularly advanced RCC remains a very aggressive and fatal disease. Several histologic subtypes of this heterogeneous tumor entity and its associated distinct molecular alterations and different clinical outcomes are described [1,2,3,4]. Three main different morphotypes of RCC exist: clear cell RCC accounts for approximately 70–80% of all RCCs, while 6–15% and 2–5% are papillary and chromophobe RCCs, respectively [5]. Clear cell RCC is the most common form of kidney cancer presenting with metastases in 30% of patients at the time of initial diagnosis [6,7]. Surgical resection (partial or radical nephrectomy) remains the principal treatment option for localized disease; nevertheless, advanced ccRCC has a poor prognosis not least because its resistance to chemotherapy and radiotherapy [8]. Despite life prolonging therapeutic improvements, such as the introduction of tyrosin, multi-kinase inhibitors (e.g., sunitinib etc.), and immunotherapeutic agents (e.g., nivolumab) [9], metastatic RCC still remains an incurable disease in almost all patients [10]. Hence, it is of paramount interest to identify novel therapeutic strategies in the context of increasingly personalized cancer care. The discovery of microRNA (miRNA) signatures in various cancerous tissues has provided novel diagnostic and prognostic insights [11,12]. MiRNAs are a class of small, endogenous, non-coding RNAs that play a crucial role in post-transcriptional regulation of their target mRNAs [13]. Non-coding RNAs in general and especially miRNAs might serve as promising biomarkers for the diagnosis, prognosis, therapy efficacy prediction, as well as act as novel RCC drug targets themselves [14,15,16,17].

## 2. Von Hippel–Lindau/Hypoxia Inducible Factor (HIF)

**Figure 1:**
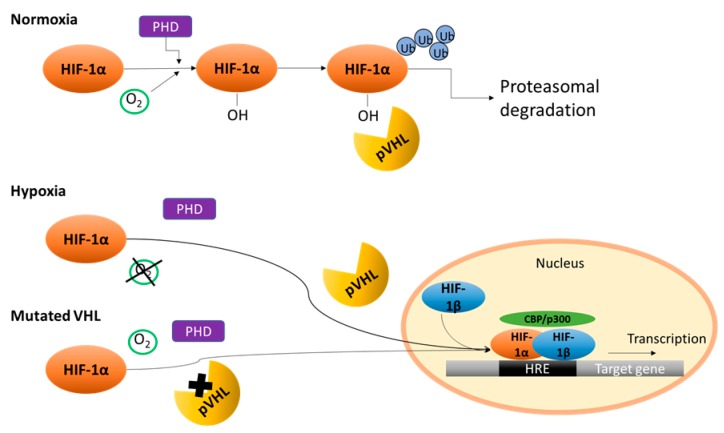
Regulation of HIF1α under normoxia and hypoxia conditions. Under normoxia conditions, the prolyl hydroxylase domain (PHD) hydroxylates HIF1α via molecular oxygen. Hydroxylated HIF1α becomes polyubiquitylated (Ub) for proteosomal degradation by binding the Von Hippel–Lindau protein (pVHL). Under hypoxic conditions, the activity of PHD is reduced. HIF1α is directly translocated to the nucleus where it binds HIF1β and CREB binding protein/p300 (CBP/p300) at the hypoxia response element (HRE) to act as transcription factor. If the VHL gene is mutated, it is not capable to bind HIF1α. HIF1α leads to the transcription of the target genes as described under hypoxia conditions.

The genetic association of RCC with the Von Hippel–Lindau (VHL) gene due to chromosomal loss in 3p25-26 was discovered in 1993 in hereditary kidney cancer families [18]. Alterations of this gene occur in the majority of clear cell RCC through mutations, deletions, and/or hypermethylation of its promoter region [19]. Mutations of VHL were also found in 60–80% of sporadic RCC cases [20]. The loss of VHL function leads to a constitutional activation of the hypoxia inducible factor (HIF) pathway. Subsequently, missing proteasomal degradation of HIFα results in increased expression of angiogenic and growth factors, including vascular endothelial growth factor (VEGF) and platelet-derived growth factor B chain (PDGF-B), that contribute to the growth and proliferation of RCC tumor cells [21,22]. The hypoxia inducible factor-1 (HIF1) is a heterodimer with an HIF1α and an HIF1β subunit. HIF1β represents the major activated transcriptional factor in response to hypoxia. Although HIF1β is constitutively expressed, the protein level of HIF1α is tightly regulated by the oxygen level. When adequate oxygen levels are reached, HIF1α is hydroxylated by prolyl hydroxylase proteins (PHDs). It is then recognized by the VHL tumor suppressor, which is an E3 ubiquitin protein ligase for ubiquitin-mediated degradation [23]. Because of the low hydroxylase activity of PHDs under hypoxia, HIF1α is stabilized and dimerized with HIF1β to form HIF1, which in turn is translocated to the nucleus. The heterodimer HIF1 activates in combination with the coactivator protein CREB binding protein/p300 the transcriptional activity of target genes within the hypoxia response element [24,25] (Figure 1).

Most cancer cells rely on aerobic glycolysis—a phenomenon called the “Warburg effect” [1,2]. Several glycolytic enzymes, such as glucose transporter isoform 1 (GLUT1), hexokinase 2, and lactate dehydrogenase A, show an elevated expression in many types of human cancer [3,4]. HIF1α is the key mediator of cellular adaption of oxygen stress and acts as a transcription factor for these genes [26]. HIF is also responsible for upregulation of pro-angiogenic genes like epidermal growth factor, VEGF, PDGF, and other proangiogenic factors, resulting in enhanced blood vessel growth [27]. In summary, the HIF pathway plays an important role as a possible driver in VHL mutant clear cell RCC. 

## 3. Angiogenesis in Renal Cell Carcinoma (RCC)

Histopathological evaluations of RCC have shown that highly vascularized neoplasms evolve out of abundant angiogenesis and abnormal blood vessel development [28]. Angiogenesis is a process that has been shown to be important in embryonic development, tissue growth, as well as in wound healing [29]. It is controlled by a balance of factors that promote or inhibit angiogenesis [30]. In RCC cells, frequent inactivation of the VHL gene contributes to HIF expression, which accelerates angiogenesis through the transcription of its target genes. During the process of angiogenesis, several growth factors interact with endothelial cells. The growth factors most frequently mentioned in the literature are the fibroblast growth factor (FGF), VEGF, transforming growth factor β (TGF-β), and cytokines as well as nitric oxide to form new vessels [31,32,33]. Angiogenic stimuli are released by tumor cells, stromal cells, as well as inflammatory cells and are recruited to the tumor site [34]. Several recent studies were able to demonstrate a strong link between miRNAs and angiogenic growth factors. For example, Zhu et al. described a miRNA called miR-146a, whose overexpression leads to an upregulation of angiogenesis and cytokine activity-associated genes, including FGF2 by directly targeting cyclic AMP-responsive element-binding protein 3-like 1 (CREB3L1). CREB3L1 functions as an inhibitor of fibroblast growth factor binding protein 1 (FGFBP1) by binding to two cyclic adenosine monophosphate (AMP)-responsive element-like sites. It prevents the expression of FGFBP1, which acts as a chaperone molecule to positively modulate the biological activities of autocrine FGF, thus supporting tumor growth and angiogenesis [35]. Hence, angiogenesis might represent a therapeutic target for the treatment of various human diseases. Moreover, the promotion of angiogenesis might be used to treat cardiovascular heart diseases, whereas anti-angiogenesis treatments might provide benefits in various tumor therapies, in particular in RCC [36,37].

## 4. Current Anti-Angiogenic Therapy

In recent years, a better understanding of the mechanisms of angiogenesis in the pathogenesis of RCC led to the development of a number of targeted therapies in the metastatic setting. The VEGF pathway plays a crucial role in tumor vascular development [36]. Its inhibition is used as one of the primary therapeutic approaches for metastatic RCC. One of these anti-angiogenic agents is bevacizumab, a VEGF-targeted monoclonal antibody that blocks the binding of VEGF-A to its receptor. Other agents, such as sunitinib, pazopanib, sorafenib, lenvatinib, and axitinib, act as inhibitors of the tyrosine kinase activity of the intracellular domain of the VEGF receptor [38]. Axitinib as a second-line treatment for metastatic RCC is a selective and second-generation multitarget TKI that inhibits the receptors VEGFR1-3. Its effectiveness may be due to the co-inhibition of further enzymes like Kit and PDGFR, which promote cell growth and proliferation [39,40].

Another call of therapeutic agents to treat metastasized RCC exhibits anti-tumor effects by inhibition of the mammalian target of rapamycin (mTOR). This pathway affects cellular functions including cell growth, proliferation, metabolism, and angiogenesis [41]. Temsirolimus and everolimus are analogues of rapamycin, which have been applied in the treatment of various solid, as well as hematologic malignancies [42,43]. In spite of directly affecting the mTOR kinase, temsirolismus and everolismus bind to FK-binding protein 12, an intracellular immunophilin, resulting in an inhibition of the kinase activity of mTOR complex 1 [44,45]. These agents have proven limited efficacy in metastasized RCC [46]. Due to an almost certain occurrence of resistances towards these agents over time, combined or novel therapeutic approaches in advanced RCC are strongly warranted [47].

## 5. MiRNA Biogenesis and Function

MiRNAs are endogenous small regulatory non-coding RNA molecules of around ∼22 nucleotides in length that regulate the activity of specific mRNA targets and play an important role in a wide range of physiological and pathological processes [48,49,50].

**Figure 2:**
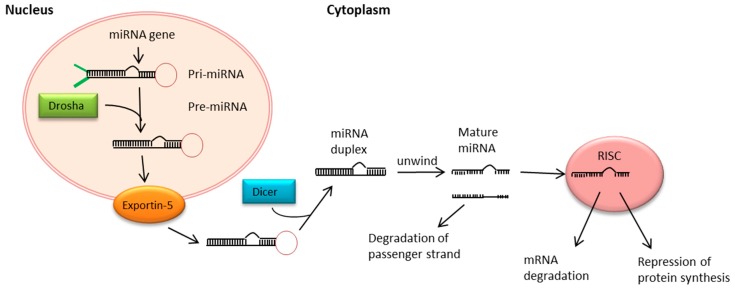
MicroRNA biogenesis and processing pathway. Arrows are indicating the way how microRNAs are processed and how they influence protein expression. Primary-miRNA (pri-miRNA) is transcribed from DNA and cleaved into precursor-miRNA (pre-miRNA), which is exported into the cytoplasm by Exportin-5 for further processing. The mature microRNA (miRNA) strand is assembled into the RNA-induced silencing complex (RISC) to bind mRNA and negatively regulates gene expression by mRNA degradation or by repression of the protein translation (as shown by the arrows pointing away from RISC respectively).

The processing of miRNAs starts with the transcription into a long primary nuclear pri-miRNA, regulated by polymerase II or RNA polymerase III. By the Drosha/DGCR8 complex pri-miRNA will be cleaved to a 70–80 bp long pre-miRNA and is then exported into the cytoplasm via exportin-5/RanGTP [51,52]. After cleaving the pre-miRNA to double-stranded mature miRNA by the RNAse III Dicer, the mature miRNA is separated into two strands: the miRNA-guide strand and the miRNA-passenger strand, which will be degraded. The guide strand is loaded into a silencing complex called RNA-induced silencing complex and is transported to the target mRNA [51,53] (Figure 2).

MiRNAs function as post-transcriptional regulators of their target genes by inducing mRNA degradation or translation inhibition, through binding to complementary nucleotides in the 3′UTR region of target mRNAs. The miRNA can be released into the cytoplasm from normal or tumor tissue, where their expression in serum and plasma is relatively stable [54,55,56]. A dysregulation of miRNAs has been extensively implicated in cancer pathogenesis in various tumor types [56]. It has been shown that miRNAs are involved in the pathogenesis of solid tumors and can act either as tumor promotor (“oncoMiR”) or tumor suppressive miRNA [57,58]. The following paragraphs will discuss several miRNAs that have been experimentally proven to be associated with the VHL gene and might serve as novel drug targets in RCC (Table 1).

## 6. MiRNAs Associated with VHL in Clear Cell RCC

### 6.1. MiR-30c

MiR-30c is involved in many biologic events, including cell apoptosis, growth and differentiation [59]. In a recent study of Huang et al. a downregulation of miR-30c in RCC by using microarray analysis was shown. Through quantitative RT-PCR analysis, a downregulation in 32 pairs of RCC tissues was accomplished, whereby 23 were tested positive. Furthermore, the authors observed a reduction of miR-30c in three RCC-cell lines after culturing under hypoxia conditions for 24 h: ACHN, Caki-1, A498 and 786-O cells. By transfecting siRNAs against HIF1/-2a, they rescued the downregulation of miR-30c. These results suggested that hypoxia might be responsible for the downregulation of miR-30c in RCC in an HIF-dependent manner. In addition, miR-30c revealed a correlation with VHL. Lower levels of miR-30c in VHL-deficient RCC cell lines A498 and 786-O were experimentally determined, indicating that VHL might regulate miR-30c expression. Huang et al. transfected Flag-VHL, a VHL expressing plasmid, into VHL-deficient cells to examine miR-30c expression. They were able to show an increase of miR-30c expression in FlagVHL-transfected A498 and 786-O cells compared to their control group. A mutation or loss of VHL in 12 of 32 RCC tissues and a significant correlation between miR-30c expression and VHL status in RCC tissues was also demonstrated. Since the loss of VHL results in an epithelial–mesenchymal transition (EMT), the downregulation of miR-30c might affect it. Through Western blot analysis Huang et al. showed a significant inhibition of E-cadherin by miR-30c downregulation in ACHN and Caki-1 cells, whereas *α-SMA* and vimentin were highly induced. The transwell assay showed promoted migration of RCC cells after inhibition of miR-30c. In conclusion, these results indicate that downregulation of miR-30c promotes EMT in RCC cells. Moreover, the authors investigated the impact of miR-30c overexpression on EMT and were able to detect an increased expression of E-cadherin. The expression of *α-SMA* and vimentin were inhibited in A498 and 786-O cells. Taken together, these results demonstrated that a high expression of miR-30c could inhibit EMT of RCC cells and therefore might prevent the spread of RCC cells from the primary tumor into the blood stream [60]. MiR-30c might be used as a first therapeutic approach at an early time stage of RCC to sustain the expression of E-cadherin, inhibiting EMT.

### 6.2. MiR-182-5p

Fan et al. demonstrated that Dicer inhibits the expression of *HIF2α*, which is a direct target of Dicer-dependent miR-182-5p in VHL-deficient clear cell RCC [61]. The enzyme Dicer is responsible for processing pre-miRNAs into their mature forms [62]. The authors demonstrated that the levels of pre-miRNA were increased in VHL-deficient clear cell RCCs in contrast to mature miRNA. There is a strong body of evidence that the enzyme Dicer is downregulated by VHL deficiency in clear cell RCC. A correlation between the enzyme and HIF2α has already been described. An overexpression of Dicer leads to reduced levels of *HIF2α* mRNA and protein, as well as the downstream target genes *VEGFA* and *GLUT-1*. In the VHL-deficient clear cell RCC cell line, OS-RC-2, that stably expresses both *HIF1α* and *HIF2α*, *HIF1α* levels were not affected by Dicer overexpression. Dicer knockdown following transfection of short hairpin Dicer (shDicer) RNA into OS-RC-2 cells upregulated *HIF2α*, *VEGFA*, and *GLUT-1* expression. Fan et al. [63] performed the same overexpression and knockdown experiment in 786-O cells, with restored pVHL levels and confirmed that Dicer suppresses *HIF2α* expression only in VHL-deficient clear cell RCCs compared to wild-type VHL clear cell RCC cells. The levels of mature miR-182-5p, a direct target of HIF2α, were decreased and the levels of the precursor molecule were increased in pVHL knockdown Caki-1 cells compared to wild type VHL cells. Furthermore, the authors were able to show an association between HIF2α and miR-182-5p, indicating an inhibition of the *HIF2α* expression in clear cell RCC cells through this Dicer-dependent miRNA. The overexpression of Dicer also has a suppressing influence on tumor growth and angiogenesis in VHL-deficient clear cell RCCs by reducing *HIF2α* expression, demonstrated in vivo and in vitro. In conclusion, Fan et al. demonstrated that reduced Dicer levels predict poor survival in VHL-deficient clear cell RCC patients [61]. According to this methodological approach, the enzyme Dicer could be used as a supporting agent in combined therapies to reduce tumor-promoting factors like HIF2α. Thus, the expression of HIF2α associated targets (VEGFA and others) responsible for angiogenesis might be limited in VHL-deficient clear cell RCC.

### 6.3. MiR-92a/ MiR-210

Valera et al. showed that 786-O and UOK117 tumor cell lines with VHL mutations had low transcript levels of *VHL* mRNA compared to two normal kidney cell lines they identified on the VHL gene. Higher levels of miR-92a were seen in higher-grade clear cell RCCs. Since the increased amount of miR-92a in clear cell RCC and decreased levels of *VHL* mRNA, the authors assume that miRNAs may influence the transcript abundance of protein-coding target genes in RCC [63].

Furthermore, they examined the activation of hypoxia in clear cell RCC. MiR-210 is upregulated by HIF1α in response to hypoxic conditions and its overexpression is associated with poor cancer-specific survival (CSS) following RCC resection [64]. Valera et al. tested whether the hypoxia-induced miRNA-210 is part of the clear cell RCC phenotype. Clear cell tumors, compared with tumors of non-clear cell histology, had significantly higher miR-210 expression levels. Mutations of VHL in exon 1, deletions, and stop mutations, as well as multiple (more than one) mutations, increased miR-210 expression. Similarly, a higher miR-210 expression was seen in high-grade tumors and in lesions with positive lymph nodes [63]. Both miR-92a and miR-210 might play an important role in high-grade tumors. The downregulation of these miRNAs could be a promising approach due to the resistance of advanced clear cell RCC against currently used agents. 

### 6.4. MiR-17-5p/MiR-224

HIF1 and VHL are predicted to be direct targets of miR-17-5p. Lichner et al. confirmed this hypothesis by Western blot analysis, the luciferase interaction assay, and qRT-PCR. Several factors that play a crucial role in clear cell RCC, such as hypoxia-inducible factor 1-α inhibitor (HIF1AN), Egl nine homolog 3 (EGLN3), VEGF-A, phosphatidylinositol-3-kinase (PI3K), phosphatase and tensin homolog, and mitogen-activated protein kinase kinase kinase 1 (MAP3K1), might be potential targets of miR-17-5p. VEGF-A is a target of the HIF1α/β transcription factor complex that induces signaling through the MAP3K1 and PI3K pathways. HIF1AN and EGLN3 are regulators of the ubiquitin ligase activity of the VHL-Cullin-RING complex [65].

Another miRNA that is dysregulated in clear cell RCC and other types of cancer is miR-224 [66]. It showed a 21.06-fold overexpression in RCC tumor tissue samples compared to adjacent, healthy kidney cortex [65]. This miRNA is already known to provide oncogenic potential in many types of cancer, such as ovarian cancer or for regulating TGF-mediated proliferation of granulosa cells [67]. However, little is known about the involvement of miR-224 in hypoxia and RCC. After treatment of SKOV-3 cells with miR-244, Lichner et al. observed a significant decrease of VHL and HIF1α protein levels. According to bioinformatic analysis, VHL is likely to be a direct target, whereas HIF1α raises the possibility to be regulated indirectly. Lichner et al. also demonstrated that miR-224 regulates the TGF-β pathway by inhibiting *SMAD4/5* [65]. Dysregulation of miR-17-5p and miR-224 might influence many pathways by targeting several factors, which lead to cancer development and tumor progression. Affecting different pathways at the same time might represent a supportive treatment modality for clear cell RCC patients.

### 6.5. MiR-28-5p

Chromosomal instability often results from defects in the mitotic checkpoint [68]. The central component herein is the mitotic spindle checkpoint protein (Mad2), a mitotic checkpoint protein, which prevents cells with incompletely assembled spindles from leaving mitosis. Both increased and decreased levels of *Mad2* can lead to tumor progression in consequence of chromosomal instability [69,70]. Hell et al. characterized the miRNA miR-28-5p as regulator of Mad2 by binding to specific target sequences in the 3′UTR region. This mechanism is triggered by the inactivation of VHL. It has been shown that in VHL-positive cancer cells, an overexpression of miR-28-5p reduced *Mad2* levels, causing chromosomal instability [71]. In contrast, the inhibition of miR-28-5p in checkpoint-deficient VHL-negative cancer cells restored *Mad2* levels. By bioinformatic analyses, the authors strengthened the hypothesis that a loss of VHL is associated with enhanced miR-28-5p expression and chromosomal instability, resulting in tumor progression in clear cell RCC [71]. Taken together, diminished levels of this miRNA would sustain chromosomal stability on the basis of functional mitotic checkpoints such as Mad2. This might provide a new therapeutic strategy to prevent clear cell RCC development and progression at the chromosomal level.

### 6.6. MiR-204

Autophagy is a survival-promoting complex to eliminate defective organelles and molecules [72]. The recycling of these intracellular constituents in lysosomes can serve as an alternative energy source during periods of metabolic stress [73]. Cancer cells use autophagy in response to metabolic stress to sustain cell viability and an increased aggressiveness [72]. Using human clear cell RCC specimens, VHL-deficient cells, and xenograft models, Mikhaylova and colleagues identified miR-204 as an inhibitor of macroautophagy, induced by VHL [74]. Thereby, MAP1LC3B (LC3B) functions as a direct target. In higher tumor grades of clear cell RCC, a decrease of miR-204 and an increase of *LC3B* levels were shown. LC3B-mediated macroautophagy seems to be necessary for RCC progression. In conclusion, VHL-induced miR-204 overexpression leads to suppression of tumor growth by targeting LC3B and therefore inhibiting macroautophagy [74]. Influencing the autophagy mechanism could significantly support currently used treatment options of clear cell RCC. Nutrient supply during periods of metabolic stress is crucial for cell survival, tumor growth, and aggressiveness [75]. For an effective tumor therapy, it is necessary to halt the intake of nutrients, which might be achieved through autophagy inhibition.

### 6.7. MiR-155

Higher expression levels of miR-155 were seen in RCC tissues compared to adjacent normal tissues [64]. Neal et al. exhibited a significant increase of miR-155 in RCC4 cells without a functional VHL gene (RCC4 − VHL compared to RCC4 + VHL). Reduced levels of miR-155 were shown through the treatment with HIF1α and HIF2α siRNAs. The induction of the miRNA in cells lacking VHL appears to be mediated mostly via HIF induction. Furthermore, miR-155 showed a weak correlation with carbonic anhydrase IX (CAIX), a transcriptional target of HIF1α, indicating that miR-155 is regulated by VHL through HIF1α [76]. The dysregulation of miR-155 might play an important role in the VHL/HIF pathway, and its downregulation could be a promising therapeutic approach for RCC, but the involvement of miR-155 in the VHL/HIF pathway remains largely unknown.

## 7. Conclusions

Several therapeutic agents, such as tyrosine kinase and mTOR inhibitors as well as immunotherapeutics (sunitinib or sorafenib), have demonstrated significant improvements regarding response rates, cancer-specific progression-free survival rates, and overall survival rates of advanced RCC patients. However, many of these patients are either primarily resistant or they develop secondary resistances towards these agents. Another issue is the possibility of increased adverse effects in combination therapies and the challenge of choosing an appropriate treatment for each RCC patient, highlighting the importance for the identification of novel prognostic markers concerning drug resistances and the development of new therapeutic strategies in RCC. Since miRNAs act as important regulators of gene expression, they show a high potential as predictive markers for the therapeutic response towards chemotherapeutic drugs. Dysregulated patterns of miRNAs appear to be tissue-specific in human cancer, and miRNAs are present in human plasma in a relatively stable form that is protected from endogenous RNase activity. Therefore, miRNA might be useful biomarkers in treatment of cancer patients. Targeting miRNAs that are involved in drug resistance mechanisms might improve the therapeutic efficacy in therapy-resistant tumor patients. It is important to find new strategies to address challenges around drug specificity, drug resistance, drug stability, and adverse effects.

## Figures and Tables

**Table 1:** MicroRNAs associated with Von Hippel–Lindau (VHL) in clear cell renal cell carcinoma (RCC). mesenchymal transition (EMT)

MicroRNA	Chromosomal Location	Tumor Suppressor/OncomiR	Proven Target Genes	Pathway Involved
MiR-30c	Unknown	Tumor suppressor	*Unknown*	Hypoxia, Epithelial-mesenchymal transition
MiR-182-5p	7q32.2	Tumor suppressor	*HIF2α*	Hypoxia
MiR-92a	Unknown	OncomiR	*Unknown*	Unknown
MiR-210	11p15.5	OncomiR	*HIF1α*	Hypoxia
MiR-17-5p	13q31.3	Unknown	*HIF1, VHL, HIF1AN, VEGFA, EGLN3, PI3K, MAP3K1*	Hypoxia
MiR-224	Xq28	OncomiR	*VHL, SMAD4/5*	Hypoxia
MiR-28-5p	3q28	OncomiR	*Mad2*	Mitotic checkpoint
MiR-204	9q21.12	Tumor suppressor	*MAP1LC3B*	Macroautophagy
MiR-155	21q21.3	OncomiR	*CAIX*	Hypoxia

## Abbreviations

RCC	renal cell carcinoma
VHL	Von Hippel Lindau
HIF	hypoxia inducible factor
miRNAs	microRNAs
VEGF	vascular endothelial growth factor
FGF	fibroblast growth factor
PDGF-B	platelet-derived growth factor B chain
TGF-β	transforming growth factor β
GLUT1	glucose transporter isoform 1
EMT	epithelial-mesenchymal transition
PI3K	phosphatidylinositol-3-kinase
CREB3L1	cyclic AMP-responsive element-binding protein 3-like 1
PHDs	prolyl hydroxylase proteins
FGFBP1	fibroblast growth factor binding protein 1
mTOR	mammalian target of rapamycin
HIF1AN	Hypoxia-inducible factor 1-alpha inhibitor
EGLN3	Egl nine homolog 3
MAP3K1	activated protein kinase kinase kinase 1
